# Editorial: A Changing Epidemic and the Rise of Opioid-Stimulant Co-Use

**DOI:** 10.3389/fpsyt.2022.918197

**Published:** 2022-07-06

**Authors:** Saeed Ahmed, Zouina Sarfraz, Azza Sarfraz

**Affiliations:** ^1^West Ridge Center for Addiction Recovery, Rutland, VT, United States; ^2^Rutland Regional Medical Center, Rutland, VT, United States; ^3^Department of Research and Publications, Fatima Jinnah Medical University, Lahore, Pakistan; ^4^Department of Pediatrics and Child Health, The Aga Khan University, Karachi, Pakistan

**Keywords:** opioid, stimulant, epidemic, COVID-19, methamphetamine, cocaine, fentanyl, overdose

## Background

The current opioid crisis in the United States has been escalating for the past two decades, and it has only worsened since the emergence of coronavirus disease in 2019 (COVID-19) (1, 2). The COVID-19 pandemic brought up unprecedented challenges in dealing with the opioid crisis, including those falling under the (i) public policy level: disruptions in addiction treatment recovery services, delivery of mental health/harm reduction services; (ii) individual level: loss of work and worsening of pre-existing psychiatric conditions; and (iii) interpersonal level: a lack of peer support, all of which may lead to increased opioid use, relapse risk, and overdoses (1, 3). The opioid epidemic and overdose deaths have been described as a “triple wave epidemic,” with the first wave involving prescription opioids, followed by heroin-related overdoses, and the current wave involving illicit fentanyl and fentanyl analogs (4). The triple wave crisis has been amplified by a “fourth wave,” which has been dominated by fentanyl but also includes cocaine and methamphetamine-related deaths (5). Although there has been a decline in overdose deaths involving prescription opioids, the opioid crisis has worsened overall (5). The growing use of synthetic opioids [such as illicitly-manufactured fentanyl (IMF)] in combination with cocaine and methamphetamine has resulted in significant increases in co-use-related overdose deaths (5).

The driving factors for stimulant use in recent years include the increasing availability of methamphetamine in the markets with the relative absence of certain opioids making the former more attractive pills (6). To some extent, restricting access to prescription opioids may be linked to an increase in methamphetamine use (6). One of the possible explanations for the rise in methamphetamine usage, according to user experience, is that it served as an opioid substitute, offered a synergistic high, and balanced out the effects of opioids in order to regain “normalcy” (6).

Although the link between opioid and stimulant concurrent use (e.g., speed-ball or goof-ball) is not novel, attention has been drawn due to an increase in the number of stimulant-related overdose deaths, most likely due to fentanyl being increasingly mixed into cocaine and methamphetamine. Concurrent use of stimulants and opioids is becoming more common, and polydrug use (e.g., co-use of a stimulant along with an opioid) has been linked to drug overdose deaths (6). Sedatives, particularly benzodiazepines or alcohol, are known to interact with opioids and are frequently implicated in opioid overdose deaths due to their respiratory depressant effect. The combination of heroin, cocaine, and injected speedballs is also a known predictor of overdose (7). Although the pharmacodynamics of stimulants in combination with opioids are not fully understood, one possible explanation for speedball-related mortality is that cocaine causes severe vasoconstriction, causing the body to use more oxygen, whereas heroin's depressant effects slow breathing rates, leading to respiratory failure. It's unclear if the link between these drugs and overdose mortality is due to drug interactions or if drug users overdose on heroin to reduce their stimulated “highs” (7). Our personal experience at a methadone clinic in rural Vermont, as well as current literature suggests that individuals who use concurrent opioids and stimulants believe stimulants are safer, combining stimulants and opioids to offset the negative effects of opioids, such as withdrawal symptoms, limiting opioid use, finding cheaper substitutes for heroin, relieving fatigue, lethargy, and some combining to enhance a “high” (6, 8). However, in many cases, fentanyl is mixed with cocaine or methamphetamine without the user's knowledge, and a person with no tolerance to opioids may suffer a fatal overdose (9, 10). The United States reported the greatest ever overdose fatalities ever recorded—totaling 93,000 deaths due to OUD (11) (Ellis et al.). Along with research efforts, recently published literature reports also underline polydrug use, which is more prevalent among individuals with OUD (Ellis et al.). As a result, the federal and state level agencies have made an effort to promote preclinical and clinical research on the effects of co-use of stimulants and opioids, as well as the development and implementation of evidence-based interventions to prevent drug overdose.

## Shifting Trends of Drug Use and Prevalence

According to the CDC, during 2015–2018, an estimated 1.6 million US adults, on average, reported past-year methamphetamine use; 52.9% of persons using methamphetamine in the past year met diagnostic criteria for methamphetamine use disorder, and nearly 25% reported injecting methamphetamine within the past year (12). The National Survey on Drug Use and Health (NSDUH) estimated that in 2019, 2 million individuals aged 12 or older used methamphetamine, up from 1.4 million in 2016 (13). Acknowledging the dearth of data on co-use, and to improve our understanding of polysubstance use among individuals with OUD, a retrospective analysis conducted from 1991 to 2020 was assessed (Ellis et al.); the authors found an 82.4% exposure to stimulants among people with OUD, whereas crack/cocaine (68.6%), prescription stimulants (50.6%), and methamphetamine (63.1%) were commonly reported. Among 7,109 individuals, the mean age of first exposure to either substance was 22.3 years. Using a national opioid surveillance system and analyzing data from 124 OUD treatment centers between 2017 and 2020, Ellis et al. report that that the average age for “initial exposure” to any stimulant or opioid has increased from 10 years to 23.5 years since the 1990s. These large shifts in populations may be linked to healthcare practitioners and systems' efforts to raise public awareness about the consequences of the medications (Ellis et al.). Therapeutic and preventive efforts should consider the newest wave's key demographics (i.e., shifting ages, rurality), poly drug use (i.e., a mixture of methamphetamine and cocaine with fentanyl), and counterfeit prescription pills.

Aside from synthetic opioids and stimulants, there has been a dramatic increase in the number of prescription and over-the-counter (OTC) drugs owing to their abuse potential at high doses or idiosyncratic methods of self-administration (14). Schifano et al. investigated the rising popularity and availability of prescription drugs (pregabalin, bupropion, venlafaxine, olanzapine, clenbuterol, and loperamide) (15). In line with this, pregabalin and gabapentin abuse appears to have increased dramatically in recent years among people with SUD, particularly those abusing opioids. Gabapentin was the tenth most commonly prescribed medication in the United States in 2016, while pregabalin ranked eighth in invoice drug spending with $4.4 billion in sales. According to the Canadian study, concomitant gabapentin and opioid exposure was associated with a 49% increased risk of dying from an opioid overdose (16), and due to such alarming rates, gabapentin is now considered an emerging threat in today's opioid epidemic. Identifying some potential gaps and challenges related to the emerging crisis of novel psychoactive substances, Schifano et al. identify the potential factors influencing this rapidly shifting drug scenario (15). For example, web-based pro-drug information to vulnerable subjects such as children and adolescents and psychiatric patients, failure to identify abuse or misuse potential during pre-marketing processes, and a lack of post-marketing substance abuse surveillance. Pharmacovigilance measures should be considered in cases of prescription and OTC drug abuse in order to detect, assess, understand, and prevent adverse effects or other drug-related problems.

## Opioids and Stimulants Trends in General and During COVID-19

Experiments involving human and animal subjects provide some evidence that stimulants such as amphetamine potentiate the analgesic effects of morphine (17). Psychostimulant drugs in animal studies present with intrinsic analgesic properties and also enhance the analgesic properties of opioids, which may explain the user groups' reasoning. Deaths involving cocaine and psychostimulants have increased in recent years, particularly among opioid users. In 2017, opioids were involved in nearly three-fourths of cocaine-related deaths and nearly half of psychostimulant-related deaths (18). Ellis et al. reported an increase in past-month methamphetamine use among opioid-dependent individuals, from 18.8% in 2011 to 34.2% (6). A recent study analyzing data from the 2015–2019 National Surveys on Drug Use and Health (NSDUH) reported that among those reporting past month heroin usage, methamphetamine use increased nearly 5-fold (from 9 to 44%). Similarly, those who used heroin in the past year used methamphetamine twice as much (22.5 to 46.7%). Rurality, past year injection drug use, and serious mental illness have all been linked to methamphetamine use among individuals who use heroin (19). The use of stimulants alone or in combination with other drugs has been linked to several social, mental, and physical health problems such as homelessness, drug-related crime, overdoses, suicide, cardiovascular diseases, and infectious disease transmission (e.g., Hepatitis C and HIV) (20).

The global prevalence of stimulants has drastically risen since 2010. Over 5 million Americans reported current cocaine use in 2020, while over 2.5 million Americans aged 12 and older reported using methamphetamine in the previous year. According to recent estimates, there are ~18 million cocaine users worldwide, with the highest rates in the US (2.1%). A recent study identified that areas dense in black and Hispanic racial/ethnic groups had a 575% increase in cocaine and opioid mortality rate compared to a 184% increase across white groups (8). From 2015 until 2019, psychostimulants' overdose among US adults, largely methamphetamine, increased by 180% (from 5526 to 15,489 overdosage estimates) (20). A rise in psychostimulant-related mortality could be attributed to increased availability and market expansion in areas and user groups traditionally associated with methamphetamine use (10). According to reports by US federal agencies, methamphetamine availability in the United States continues to be widespread, its purity and potency remain high, and its price remains relatively low (10). In our observation working with patients, patients report an increase in stimulant use due to the growing fear of fentanyl adulteration of heroin and the risk of overdose.

The COVID-19 pandemic has had an impact on the network of cocaine trafficking supply lines, but it has not significantly reduced overall supply to the United States. Despite the rising crisis, there are no FDA-approved pharmacological treatments to treat amphetamine or cocaine addiction, and unfortunately no antidotes to treat stimulant overdoses. During the COVID-19 era, the US, saw a rise in overdose deaths caused by illicit fentanyl, fentanyl analogs, methamphetamine, and cocaine, often in combination or mixed with other drugs, evidenced by the alarming 100,306 drug overdose deaths in the US, during the 12 months ending in April 2021, a 28.5% increase from the 78,056 deaths during the same period the year before (21). The main drivers were synthetic opioids (i.e., fentanyl), but stimulants like cocaine and methamphetamine were also increased. During the pandemic, drug use increased in both quantity and frequency, leading to an increase in drug overdose deaths. A plausible explanation for the increase in substance use during the pandemic included coping with emotional stress, social isolation, economic stress related to COVID-19, and increased general anxiety and depression. The rising number of overdose deaths involving synthetic opioids could be attributed to heroin shortages and the economic downturn during the pandemic, which led to users switching to substances such as low-cost fentanyl and its derivatives.

The disruption in heroin supply has also exacerbated harmful drug use, such as the use of home-produced injectable opioids like “krokodil,” also known as “Russian Magic,” a cheap but extremely dangerous substitute for heroin. During the peak of the pandemic, we saw two cases at our clinic who reported using krokodil in the absence of heroin. Contrary to the majority of reports indicating a significant increase in drug use and overdose deaths around the world, an Italian study assessed the psychopathological burden in people with substance use disorders, more specifically craving changes in daily habits, which only showed modest change during the COVID-19 pandemic (22). The Italian cohort posits that craving for drugs is considered as a significant therapeutic target for lowering the risk of relapse and improving patients' quality of life (22). Low levels of craving during a pandemic like COVID-19 may be attributable to a perceived lack of availability of the drugs and reduced societal pressure on people using drugs. The study found that craving was lower in inpatients than in outpatients, highlighting the importance of residential treatment in substance use disorders (22). The findings of this study could be put into practice as one of many options for dealing with the 4th wave crisis.

## Recommendations and Future Directions

In light of the many risks and consequences of stimulant and opioid use, some interventions can reduce the rate of fatal opioid overdoses. For example, (i) the scalability of rapid fentanyl test strips to detect fentanyl in illicit drugs may be useful in harm reduction interventions (23). (ii) Initiating or continuing medications for opioid use disorder. (iii) Due to the rise in polydrug overdoses, treatment providers must assess for concurrent substance use disorders and offer evidence-based treatments. (iv) Distribution of naloxone through cost-effective, pharmacy- and community-based programs; expanding the locations of naloxone distribution centers, particularly in minority populations, rural communities, and homeless shelters. (v) Naloxone is not effective against stimulant overdose, but it should be offered due to the rise of concurrent opioid use. (vi) Educating individuals not to use drugs alone, ensuring that naloxone is available and that people who use drugs and their loved ones know how to use it. (vii) Educating individuals that they may require repeated doses of naloxone to reverse an overdose due to the potency of IMF and fentanyl analogs (24). (viii) On a personal level, individuals exposed to opioids and stimulants together ought to be aware of symptoms, pulse rate, heart rate, and rhythm for prevention (25). (ix) Although there are no FDA-approved medications to treat stimulant use disorders, treatment providers offer evidence-based treatment approaches such as community reinforcement, motivational interviewing, and cognitive-behavioral therapy combined with contingency management (12). (x) identifying jurisdictions and vulnerable groups (e.g., IV drug users) who are at higher risk of infectious disease, and expanding harm reduction approaches for those groups, e.g., syringe exchange programs (SEPs). (xi) With the growing COVID-19 and opioid epidemic, wearable monitors with inbuilt artificial intelligence-powered sensors linked to medical devices can become key in attaining urgent medical attention (26). This growing problem of stimulant-opioid co-use requires an emphasis on access to evidence-based treatment.

This paper serves as a call to action for the high prevalence of substance and opioid co-use, albeit with a paucity of data (Ware et al.). Polydrug use develops due to various reasons such as accessibility, motivation, and awareness. The fourth wave of the opioid crisis has been on the rise since the late 20th century, however, with the COVID-19 pandemic in the mix, demographic shifts from the adults to the pediatric population may reemerge with shifting behaviors (27). The recent opioid epidemic surge provides an opportunity to understand the conducive factors to polydrug use and OUD amplified by the COVID pandemic. In the broader sphere of addiction medicine and public health, educational and preventive efforts are required to reduce harmful outcomes and promote treatment to reduce morbidity and mortality trends, paralleled with narcotics regulations and monitoring; an understanding of the social narrative and socioeconomic influences on substance consumption is essential to effectively address the “fourth wave of the opioid crisis.”

## Author Contributions

SA, ZS, and AS contributed to the conceptualization, planning and implementation, writing—original draft, and review—of the research. SA supervised this article, conducted analysis and interpretation of literature review, and critical revision of the manuscript. All authors contributed to the article and approved the submitted version.

## Conflict of Interest

The authors declare that the research was conducted in the absence of any commercial or financial relationships that could be construed as a potential conflict of interest.

## Publisher's Note

All claims expressed in this article are solely those of the authors and do not necessarily represent those of their affiliated organizations, or those of the publisher, the editors and the reviewers. Any product that may be evaluated in this article, or claim that may be made by its manufacturer, is not guaranteed or endorsed by the publisher.
